# The Index of Cognitive Activity as a Measure of Cognitive Processing Load in Dual Task Settings

**DOI:** 10.3389/fpsyg.2018.02276

**Published:** 2018-11-30

**Authors:** Jorrig Vogels, Vera Demberg, Jutta Kray

**Affiliations:** ^1^Center for Language and Cognition, University of Groningen, Groningen, Netherlands; ^2^Department of Computational Linguistics and Phonetics, Saarland University, Saarbrücken, Germany; ^3^Department of Mathematics and Computer Science, Saarland University, Saarbrücken, Germany; ^4^Department of Psychology, Saarland University, Saarbrücken, Germany

**Keywords:** ICA, cognitive load, dual tasking, language comprehension, pupil size, simulated driving

## Abstract

Increases in pupil size have long been used as an indicator of cognitive load. Recently, the Index of Cognitive Activity (ICA), a novel pupillometric measure has received increased attention. The ICA measures the frequency of rapid pupil dilations, and is an interesting complementary measure to overall pupil size because it disentangles the pupil response to cognitive activity from effects of light input. As such, it has been evaluated as a useful measure of processing load in dual task settings coordinating language comprehension and driving. However, the cognitive underpinnings of pupillometry, and any differences between rapid small dilations as measured by the ICA and overall effects on pupil size are still poorly understood. Earlier work has observed that the ICA and overall pupil size may not always behave in the same way, reporting an increase in overall pupil size but decrease in ICA in a dual task setting. To further investigate this, we systematically tested two new dual-task combinations, combining both language comprehension and simulated driving with a memory task. Our findings confirm that more difficult linguistic processing is reflected in a larger ICA. More importantly, however, the dual task settings did not result in an increase in the ICA as compared to the single task, and, consistent with earlier findings, showed a significant decrease with a more difficult secondary task. This contrasts with our findings for pupil size, which showed an increase with greater secondary task difficulty in both tasks. Our results are compatible with the idea that although both pupillometry measures are indicators of cognitive load, they reflect different cognitive and neuronal processes in dual task situations.

## Introduction

The relative size of the pupil has been an established measure of processing load in cognitive tasks such as memory recall and language comprehension for over 50 years (e.g., Kahneman and Beatty, 1966; Just and Carpenter, 1993). According to a common definition, cognitive load quantifies “how hard a cognitive system needs to work to perform a given task” (Just et al., 2003). Under this definition, cognitive load is related to the amount of attentional or working memory (WM) resources needed to solve a task. This is always relative to the total capacity: People with a limited WM capacity will experience a higher load for the same amount of resources used. The source of the load is thereby not relevant: Cognitive load can either have an intrinsic source, such as the novelty of the stimulus (e.g., high or low predictability), or an extrinsic source, such as an externally imposed workload (e.g., a dual task; Just and Carpenter, 1993).

Larger dilations of the pupil are thus considered to correspond to greater cognitive load during task performance. However, using the size of the pupil as a measure of cognitive load suffers a few drawbacks. Firstly, the pupil also dilates and contracts as a function of the amount of light input. This means that the luminosity of the visual scene needs to be carefully controlled to avoid confounds between dilations due to cognitive activity and the light reflex.^1^ Secondly, the overall dilation and contraction of the pupil is too slow to accurately capture the cognitive response to stimuli that succeed each other rapidly or that overlap. This may be problematic for tasks such as driving, where continuous steering movements are necessary (see Demberg et al., 2013a), spoken language comprehension, where the rate of word presentation is very high (e.g., Hyönä et al., 1995), and dual tasks (Demberg, 2013). To overcome these drawbacks, a different pupillometric measure of cognitive load, the Index of Cognitive Activity (ICA; Marshall, 2000), has been developed, which until recently has received little attention. The ICA is calculated from the overall pupil size by counting the number of rapid increases in pupil size within a certain time period (for a detailed description, see Marshall, 2002; Demberg, 2013). It disentangles rapid dilation changes due to cognitive activity from slow changes due to differences in light input, by separating high and low frequency components from the signal. In addition, the ICA has a shorter latency than overall pupil size, and a low auto-correlation. A low auto-correlation means that the frequency of rapid dilations in a certain time frame shows very little correlation with the frequency of rapid dilations in a previous time frame (Demberg and Sayeed, 2016). Both its shorter latency and its low auto-correlation make the ICA particularly suitable for experiments where high time resolution is beneficial for relating a measured effect to the stimulus that caused it (see Demberg and Sayeed, 2016). Because it can be obtained using a conventional (remote) eye-tracking device, the ICA is also more easily measured in naturalistic tasks, such as driving, than other fine-grained but more cumbersome physiological measures of cognitive load, such as EEG, heart rate, or skin conductance.

In sum, the existing evidence suggests that the ICA can be used as a reliable pupillary measure of cognitive load, and that it is complementary to the conventional pupil size measure, in tasks that require more fine-grained analyses or in which lighting conditions cannot be held constant. What is not yet clear, however, is whether the ICA is equivalent to overall pupil size in terms of its cognitive underpinnings, or whether the two measures may tap into different cognitive processes. In particular, little is known about how the ICA reacts to cognitive load caused by performing multiple tasks at the same time. The first aim of the present study is therefore to systematically examine whether the ICA is a sensitive measure of processing load due to a dual task. If so, it can serve as a useful psychophysiological indicator of processing difficulty in naturalistic settings, such as listening to speech while driving. Ultimately, more insight in people’s processing difficulties in various situational contexts will benefit, for example, the development of more adaptive natural language generation applications to avoid cognitive overload, such as in-car dialog systems that adapt linguistic complexity to individual users (e.g., younger or older adults) and different traffic situations. A second goal of this study is to clarify how the ICA relates to traditional pupil size measures, and thus to gain more insight in its interpretation. Before presenting the current study, we first provide a brief summary of what is currently known about the neurological mechanisms underlying pupil dilations.

Recent research has led to a better understanding of the neurological underpinnings of pupil dilations. It has been found that pupil dilations are closely correlated with the release of norepinephrine (NE) from the locus coeruleus (LC) area in the brain stem, which amplifies neuronal firing and enhances neuron synchronization, and so helps stimulus processing. In addition, two modes of LC activity have been identified (Aston-Jones and Cohen, 2005; Gilzenrat et al., 2010): a tonic mode, which represents the baseline firing rate of the LC (0–5 Hz), and a phasic mode, which is characterized by brief, rapid increases in firing rate (up to 20 Hz). High levels of baseline firing (tonic mode) are associated with diffuse focus of attention and with an increased ability to detect new stimuli (Laeng et al., 2012). This mode is therefore also called the explorative mode. The phasic mode, also called the exploitative mode, is associated with increased focus of attention to subjectively salient and task-relevant stimuli, where ‘salience’ could refer to, for instance, novelty (surprisal) or complexity (e.g., Nieuwenhuis et al., 2005; Alnæs et al., 2014). The phasic neuronal firings help focus attention on the stimulus, process it, and lead to better task performance. An increase in phasic LC activity has also been linked to a greater amplitude of the P3 ERP component, which is associated with attending to and processing of unexpected or task-relevant stimuli (the LC-P3 hypothesis; Nieuwenhuis et al., 2005; Murphy et al., 2011).

According to the Adaptive Gain Theory (Aston-Jones and Cohen, 2005), LC activity can adaptively shift to a different mode (from tonic to phasic or vice versa) as task requirements change, to increase the gain (neural responsiveness) in those brain areas that are relevant for the current task (either exploiting or exploring). If attentional focus is needed on a specific task, the LC will respond with a higher frequency of phasic activity; if the task is resolved and the brain needs to be open for exploring new inputs, there will be a switch to tonic LC activity. Hence, assuming that rapid pupil dilations are directly related to rapid phasic increases in firing rate in the LC, an increase in the frequency of rapid pupil dilations, and thus in the ICA, should correspond to the invocation of additional attentional resources to focus on the task at hand.

Empirical evidence supports the ICA as a reliable measure of cognitive load in a variety of cognitive tasks. For example, the frequency of ICA events (rapid pupil dilations) has been shown to increase with higher task difficulty in digit span tasks, visual search tasks, simulated driving and language processing tasks (Marshall, 2002, 2007; Schwalm et al., 2008; Demberg and Sayeed, 2016). The ICA also seems sensitive to strategic shifts in behavior in response to changing task demands (Marshall et al., 2003; Schwalm et al., 2008; Hampson et al., 2010). Recently, the ICA has been successfully applied to Visual World studies in combination with eye-tracking, in which linguistic reference to unexpected objects in the visual scene resulted in a higher ICA value, as well as to more eye fixations on those objects (Demberg and Sayeed, 2016; Sekicki and Staudte, 2017; Tourtouri et al., 2017). Furthermore, Demberg and Sayeed (2016) measured the ICA response in several reading and spoken language comprehension experiments, in order to investigate its sensitivity to different manipulations of linguistic complexity in language comprehension. The results of the experiments supported their hypothesis that the ICA reflects the amount of cognitive load during sentence processing. For example, the frequency of ICA events increased with grammatical violations, semantic anomalies, and object relative clauses as compared to subject relative clauses. These effects of linguistic processing difficulty largely remained when the linguistic tasks were combined with a driving task in a dual-task setting, indicating that the presence of overlapping stimuli in a dual task need not mask language processing effects on the ICA. This underscores the fine-grained sensitivity of the ICA.

It is less clear how the addition of a secondary task *itself* affects the ICA, i.e., whether the ICA value increases in a dual task as compared to a single task. Given the general finding that driving while speaking or listening to language greatly impairs driving performance (e.g., Strayer et al., 2003; Drews et al., 2008; Charlton, 2009; Gaspar et al., 2014), one would predict that performing these tasks simultaneously increases cognitive load, and in turn leads to an increase in the frequency of ICA events. However, Demberg et al. (2013b) found that the frequency of ICA events *decreased* under dual-task conditions involving language comprehension and driving compared to single-task driving, contrary to expectation. The authors proposed that this might be due to “downsampling” of the tasks, i.e., people may have shifted their attention to one of the tasks, or allocated less attention to either task. However, overall pupil size did increase under dual-task conditions, in line with an increase in cognitive load. This suggests that the ICA, as a more dynamic measure than pupil size, may capture a different underlying cognitive process. For example, it is possible that an increase in the ICA reflects a shift to the exploitative mode in brain activity, and that the explorative mode needed to distribute focus of attention over multiple tasks is characterized by a lower level of the ICA. Still, it is unclear to what extent the decrease in the ICA in dual tasks is a general phenomenon or specific to the dual-task setting used by Demberg et al. (2013b).

To investigate how the ICA reacts to cognitive load induced by dual-task situations, and to thereby further explore the cognitive processes underlying the ICA, the present study measures the pupil’s ICA response to different manipulations of task difficulty in two previously untested dual-task settings: a listening task combined with a memory task, and a simulated driving task combined with the same memory task. In this way, we are able to infer whether the ICA responds differently to task combinations involving language comprehension than to task combinations involving driving.

Task difficulty was manipulated in two ways. The first manipulation concerned the intrinsic complexity of the task. In the listening task, we presented participants with sentences that differed in semantic surprisal: half of the sentences contained a semantically surprising element, while in the other half this element was predictable. Here, we expected to confirm the sensitivity of the ICA to different degrees of linguistic complexity, as shown in earlier studies (Demberg et al., 2013a; Engonopoulos et al., 2013; Demberg and Sayeed, 2016). Specifically, the frequency of rapid pupil dilations was predicted to increase after the presentation of a semantically surprising word as compared to an expected word. The simulated driving task had two difficulty conditions, of which the more difficult condition was assumed to create a higher load than the easier condition, and thus to result in a higher frequency of rapid pupil dilations (cf. Demberg et al., 2013b).

The second manipulation concerned the extrinsic cognitive load, which was manipulated by varying the presence or absence of a secondary memory task in which series of numbers had to be memorized and recalled. If the ICA is also sensitive to increases in extrinsic cognitive load, we would expect an increase in the frequency of ICA events in our dual tasks as compared to the single tasks. The memory task had two difficulty conditions, and we predicted dual-task effects to be stronger in the more difficult condition.

In order to investigate the ICA response to both types of cognitive load, we analyze our data from two separate perspectives: first, to investigate the sensitivity of the ICA to processing load due to the addition of a secondary task (extrinsic load), we compare the average ICA value in the dual tasks to the average ICA value in the single tasks. Second, to confirm the sensitivity of the ICA to processing load due to complex stimuli (intrinsic load), we analyze the ICA value in response to each stimulus in the listening task as well as in the memory task. In addition, we compare the stimulus-locked ICA response with pupil dilations in response to our stimuli, as well as the average ICA value within a single or dual task with the average overall pupil size during that task. Pupil dilations are expected to be larger in response to the more difficult than the easier stimuli, but as a less dynamic measure of cognitive load, pupil dilation should show slower responses than the ICA. Still, we expect overall pupil size to generally be larger in dual- than in single-task contexts, as well as in the more difficult than in the easier secondary memory task condition. Even though lighting conditions cannot be tightly controlled in simulated driving tasks in general (see also Kun et al., 2012), our specific task setup was such that luminance changes are negligible. This allows us to also analyze overall pupil size.

Finally, we also investigate performance on the three different tasks (accuracy on the comprehension questions, driving performance, and recall on the memory task). Here, we predict a drop in task performance as cognitive load increases, both due to the intrinsic and due to the extrinsic load manipulation, unless the recruitment of additional cognitive resources, as signaled by the pupillometric measures, enabled participants to retain their level of performance.

## Materials and Methods

### Participants

Thirty-two undergraduate students recruited from Saarland University (19 female; *age range* = 20 – 34 years; *mean age* = 25.0; *SD* = 3.5) participated in this study. All participants were native speakers of German and reported normal or corrected-to-normal vision. Nine participants wore glasses and six wore contact lenses. Nine participants were left eye dominant and one did not show eye dominance, as established by alternating closing each eye while looking at a dot on the wall through a hole between the hands. All but one participant possessed a driver’s license. All participants provided written informed consent in accordance with the Declaration of Helsinki using a recommended procedure approved by the ethics committee at Saarland University. Participants were paid €16 for compensation.

### Apparatus

**FIGURE 1:**
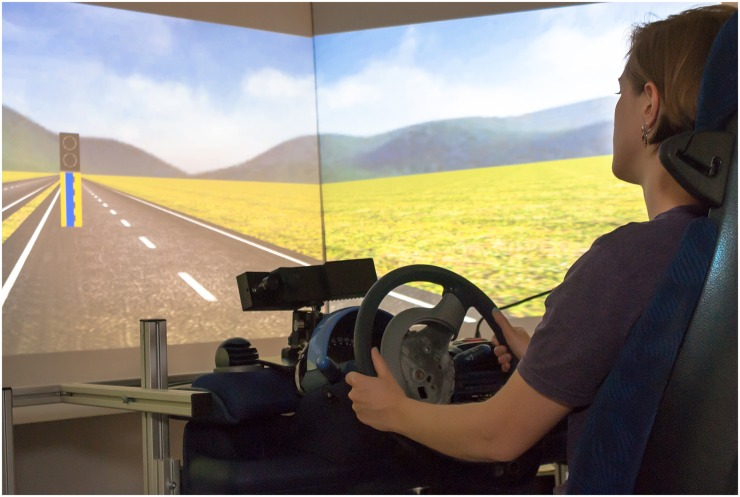
The driving simulator. Written informed consent for the publication of this image was obtained from the individual depicted.

The experiment was run in a driving simulator consisting of two front seats, dashboard, steering wheel and gas and brake pedals taken from a real car. We used the OpenDS 3.0 software^2^ to provide the driving environment, which was projected on three large panels positioned in a 180° curve around the car (see Figure 1). The driver’s seat was aligned with the center of the middle panel. An Eyelink 1000 Plus eye tracker (SR Research, Mississauga, Ontario, Canada) was placed just behind the steering wheel in order to collect pupil dilation data from both eyes, sampled at 250 Hz. The driving simulation and the presentation of the numbers for the memory task were controlled from a separate PC using the Experiment Builder software^3^, via TTL signals sent over a parallel port connection. The audio files for the listening task were also played from that PC over Creative Gigaworks T20 speakers, which were placed at the bottom right and bottom left of the driving simulator screen.

### Tasks and Stimulus Materials

#### Listening Task

The first primary task was a language comprehension task, in which participants listened to various auditorily presented sentences differing in complexity, and responded to comprehension questions about those sentences. For this task, we created 96 German sentences, converted to synthesized speech using the MaryTTS system^4^ to avoid confounds of prosody. Forty-eight sentences varied in syntactic complexity, half of them being subject relative clauses, and the other half being object relative clauses.^5^ Another forty-eight were sentences in which semantic surprisal (high or low predictability) was manipulated. Here, we created pairs of sentences in which a certain target word was highly predictable in one version, but violated a strong prediction in the other (cf. Federmeier et al., 2007; Otten and Van Berkum, 2008). Predictability was manipulated by varying only one word in each sentence pair. An example is given in (2), with the target word presented in bold.

(2)
  (a) Low surprisal
    Weil Petra für den Grillabend nichts zum Trinken hatte, kaufte sie sich ein **Bier** in einem nahegelegenen Geschäft.
    ‘Because Petra had nothing to drink for the barbecue evening, she bought herself a **beer** in a nearby store.’
  (b) High surprisal
    Weil Petra für den Grillabend nichts zum Anziehen hatte, kaufte sie sich ein **Bier** in einem nahegelegenen Geschäft.
    ‘Because Petra had nothing to wear for the barbecue evening, she bought herself a **beer** in a nearby store.’

Cloze probability of the target word was obtained using a cloze task, in which a different group of participants was asked to complete the sentences, cut off before the target word, following their first intuition. Mean cloze probability was 86% for the low surprisal versions, and 0.17% for the high surprisal versions. To control for the slight difference across the two conditions in the context preceding the target word, we also included versions in which the same contexts were matched to the other condition (e.g., *zum Trinken* ‘to drink’ and *zum Anziehen* ‘to wear’ matched to the target word *Kleid* ‘dress’). Because this resulted in four versions of each item, we created four participant lists according to a Latin square design, such that each version of a given item appeared only once on a list. In addition to the experimental items, we created 48 filler items, which included sentences similar in structure to the experimental items, but which did not trigger strong predictions for a particular word (cf. e.g., Otten and Van Berkum, 2008). The order in which the linguistic materials were presented was randomized for each participant, with the restriction that not more than two items of the same type (relative clause, semantic, and filler) appeared consecutively. Finally, 16 practice items matching the other items were created.

To assess whether participants were paying attention to the linguistic stimuli, we created yes/no comprehension questions that were auditorily presented after every other sentence, and to which participants had to respond verbally. Answers were recorded by the experimenter. For each item, the appearance of a comprehension question as well as its correct answer were counterbalanced across blocks.

#### Driving Task

The second primary task was a driving task, for which we used a modification of the Continuous Tracking and Reaction (ConTRe) task (Mahr et al., 2012). The ConTRe task is a combination of two well-known cognitive tasks: a tracking task and a reaction task. In the present study, we only used the tracking task. Here, a yellow vertical bar (the reference bar, see Figure 1) moves horizontally across a straight road, stopping at random positions for 2 s before continuing. The steering wheel controls the car’s position on the road as well as a second, blue, vertical bar (the steering bar). The driver’s task is to control the lateral position of the steering bar such that it overlaps with the yellow reference bar as much as possible. Two task difficulty conditions were created by manipulating the speed of the reference bar: In the easy condition, it moved slowly, at a speed of 1 m/s, and in the difficult condition, it moved faster, at a speed of 2.5 m/s.

#### Memory Task

In the dual-task conditions, participants had to perform a secondary memory task while performing the primary task (either the listening or the driving task). To integrate this task naturally with the context of the driving simulator, we had participants recall speed limit signs appearing on overhead bars above the road. The signs showed speed limits ranging between 10 and 90 km/h (only tens). Again, there were two difficulty conditions: In the easy condition, only two consecutive signs were shown and had to be remembered, while in the difficult condition, participants had to remember four signs. Each sign was presented for 2 s before the next sign appeared. After the presentation of the signs, a 500 ms 550 Hz beep sounded, and participants had to verbally recall the signs in the order of presentation. Responses were recorded by the experimenter. In the dual-task condition of the listening task, the speed limit signs were always presented in the same time window as the presentation of the target words. In addition, the sign recall task always preceded the comprehension question.

### Procedure

After participants had filled out a consent form and a short demographic questionnaire, they were seated in the driver’s seat of the driving simulator. First, the eye tracker was set up and calibrated. Then, the experiment started with a training phase, in which all task conditions (i.e., the two primary tasks and their combination with the memory task) were practiced in separate blocks. Instructions for each task condition were given both verbally by the experimenter and on the simulator screen before each training block. To facilitate correct eye tracking, participants were instructed to keep looking at the center of the screen as much as possible.

The actual experiment consisted of eight blocks, in which each of the four task conditions was performed twice. Block order was counterbalanced across participants. Of the four listening blocks, two were single-task blocks and two blocks were combined with the secondary memory task. Each block consisted of 12 relative clause items, 12 semantic items, and 12 fillers. Of the four driving blocks, there were also two single- and two dual-task blocks, of which one had the easy driving condition, and the other the difficult driving condition. For one single-dual task block pair, the single task block preceded the dual task block, and the reverse order was used for the other pair. After each block, participants were allowed to take a short break. The eye tracker was recalibrated before starting the next block. After the experiment, participants performed two psychometric tests to allow for the inclusion of control variables accounting for individual differences in cognitive capacity. The results of these tests are available in the Appendix. A single experimental session, including instructions, setting up the eye tracker, and the psychometric tests, took about 2 to 2.5 h.

### Experimental Design and Analyses

To summarize the experimental design, there were two primary tasks (a listening task and a driving task), which were either performed in isolation (single task) or combined with a secondary memory task (dual task). Both the primary tasks and the secondary task had an easy condition (low semantic surprisal, slow moving bar, and 2 speed limit signs, respectively) and a difficult condition (high semantic surprisal, fast moving bar, and 4 speed limit signs, respectively). Separate analyses were run on the listening task and the driving task. The listening task included Block type (single/dual) and Semantic surprisal (low/high) as within-participants factors, and the driving task included Block type (single/dual) and Driving difficulty (easy/difficult) as within-participants factors. Secondary task difficulty (2 signs/4 signs) was nested under the factor Block type, since there was only a secondary task in the dual task conditions. For both analyses, we integrated these two factors, resulting in a 3-way factor Secondary task difficulty (single/dual 2 signs/dual 4 signs).

Given a sampling rate of 250 Hz, we obtained one pupil size data point for every 4 ms. The Index of Cognitive Activity (ICA) was derived from these pupil size data by a procedure described in Marshall (2000), in which a wavelet analysis is used to decompose the raw pupil dilation signal into low-frequency components corresponding to the light reflex and high-frequency components triggered by cognitive activity. In this analysis, only rapid increases in pupil size that are large enough to exceed a threshold separating them from noise are counted as indicators of cognitive activity. The procedure was patented in 2000 (US Patent Number 6,090,051), and the ICA values can be obtained via the Cognitive Workload Module (version 3) software program (EyeTracking Inc., San Diego, CA, United States).^6^ By default, this program counts the number of rapid pupil dilations per second, and then normalizes and transforms these values (Marshall, 2000, 2007). However, to be able to investigate responses to unexpected words in the language comprehension task, a more fine-grained time scale is required. For the present study, we therefore obtained the raw ICA events (rapid pupil dilations), and then resampled them to get the number of ICA events per 100 ms (10 Hz). The pupil size data were also resampled to get one observation per 100 ms. Because the ICA cannot be calculated when there is insufficient subsequent data, we removed the last 2 s of every block.

Across all participants, we had to remove 11 blocks because of eye-tracker or driving simulator problems. In addition, missing individual data points (due to blinks, track loss) were removed from data analysis. Furthermore, gaze positions that fell outside of the driving simulator center screen were removed. Together, these steps resulted in a loss of 9.8% of the original number of data points.

For both the listening task and the driving task, we performed two separate analyses, the first focusing on the comparison between single and dual task blocks, and the second focusing on stimulus-evoked cognitive load. For the single vs. dual task analysis, we compared the frequency of ICA events and overall pupil size averaged over the single task blocks with their averages on the dual task blocks. For the dual task blocks, we also distinguished between the easy (2 signs) and the difficult (4 signs) memory task. Additionally, we controlled for effects of the primary task manipulations (either semantic surprisal or driving difficulty). Because the absolute size of the pupil is not comparable from one participant to another, the pupil size data points were centered and scaled for each participant, such that the mean pupil size was set to 0 and the standard deviation to 1. Pupil sizes smaller or larger than 2.5 SD from the mean pupil size per participant were removed, resulting in an additional loss of 2.9% of data points. For the listening task, we also analyzed the accuracy (proportion correct) of answers to the comprehension questions. For the driving task, we analyzed the average absolute steering deviation (the distance between the steering bar and the reference bar; cf. Demberg et al., 2013b). For the dual task blocks, performance on the memory task was analyzed as well (proportion of speed limit sign series recalled correctly).

For the stimulus-evoked load analysis, we investigated the sensitivity of the ICA and the dilation of the pupil to the presentation of a complex stimulus in the listening and memory tasks. For the listening task, we measured the two pupillometric responses on the presentation of a semantically surprising word as compared to an expected word, while controlling for the difficulty of the secondary task (single, dual 2 signs, dual 4 signs). For both the listening and the driving task, we also measured the pupillometric responses on the presentation of the final sign in each series of 2 and 4 signs in the memory task, controlling for primary task difficulty. The final sign was chosen on the assumption that the presentation of the last number in a series to be recalled would show the largest amount of cognitive workload. Since these analyses compare conditions within a single block, pupil size data points for each participant were centered and scaled per block, which removes variance due to eye tracker recalibration in between blocks. Pupil sizes smaller or larger than 2.5 SD from the mean pupil size per participant per block were removed, resulting in an additional loss of 3% of data points.

Previous research (Demberg and Sayeed, 2016) has revealed that the ICA effect of semantically surprising words occurs about 600–1100 ms after the target word in a self-paced reading task, and slightly earlier in a listening-driving dual task. Therefore, for our stimulus-evoked load analysis, we calculated the average ICA for both eyes within a 500 ms time window centered around 750 ms from the onset of the target word (500–1000 ms) in each semantic surprisal item, or from the onset of the presentation of the last speed limit sign in the memory task (either the second or the fourth, depending on the condition). Since changes in pupil size have a larger latency than the ICA, the target region for measuring pupil dilations was a 500 ms period centered around 1450 ms from the onset of the target word or the final sign (1200–1700 ms). This time lag was established *post hoc*, based on the latency with which changes in pupil size reacted to the reference bar in the driving task. The average pupil dilation in the target region was calculated by subtracting the average pupil size within the baseline region from the pupil size at each 100 ms time point during the target region. The baseline region was defined as the 1-s region preceding the target region (for the listening task), or as the 1-s region preceding the appearance of the first overhead bar supporting the signs (for the memory task). We additionally removed trials for which 75% or more of the data points in the target region were missing. For the listening task, this resulted in a further loss of 2.3% of data points for the ICA analysis, and 2.8% of data points for the pupil dilation analysis. For the memory task during dual task listening, it resulted in a loss of 1.9% of data points for the ICA analysis, and 2.0% of data points for the pupil dilation analysis. For the memory task in dual task driving, it resulted in a loss of 0.8% of data points for the ICA analysis, and 1.0% of data points for the pupil dilation analysis.

For both the single vs. dual task analysis and the stimulus-evoked load analysis, we fitted linear (for pupil size and driving performance) or generalized linear (for ICA, question answering accuracy and sign recall) mixed effect models to the data, using the lme4 package version 1.1.13 in R version 3.4.0 (Bates et al., 2014). All models included the fixed predictors Primary task difficulty (semantic surprisal or driving difficulty) and Secondary task difficulty. The predictor Primary task difficulty was centered to reduce collinearity. The 3-level predictor Secondary task difficulty was Helmert coded, with one binary predictor comparing single to dual tasking, and one comparing easy (2-sign) to difficult (4-sign) dual tasking. The interaction term was only included in the models if it significantly contributed to model fit according to a likelihood ratio test. Participant was added as a random intercept in all analyses, a random intercept for Eye (left/right; nested under Participant) was added in the pupillometric analyses, and a random intercept for Item was added in the listening task analyses. We attempted to fit models with a maximal random effects structure, including all random slopes (Barr et al., 2013). When a model did not converge, we simplified the random effects structure, starting with removing random correlations, and then removing random effects with the lowest variances (see Bates et al., 2015, for a description of this method). Random slopes for the principal variable(s) of interest always remained in the model. P-values for the linear mixed models were obtained via the lmerTest package (Kuznetsova et al., 2017).

## Results

In this section, we first report the results for the single vs. dual task analysis (see section “Single vs. Dual Task Analysis”), and then the results for the stimulus-evoked load analysis (see section “Stimulus-Evoked Load Analysis”). Results for the different tasks are presented separately. For the listening task analyses, data from two participants were excluded, because the removal of blocks with eye tracker problems resulted in insufficient data for these participants. For the driving task analyses, data from four additional participants were excluded, because the removal of blocks with eye tracker problems or driving simulator failure resulted in insufficient data for these participants.

### Single vs. Dual Task Analysis

For the single vs. dual task analysis, we report results for the ICA, overall pupil size, and performance on both the primary and the secondary task, for the listening and the driving task separately.

#### Listening Task

##### Index of cognitive activity (ICA)

**FIGURE 2:**
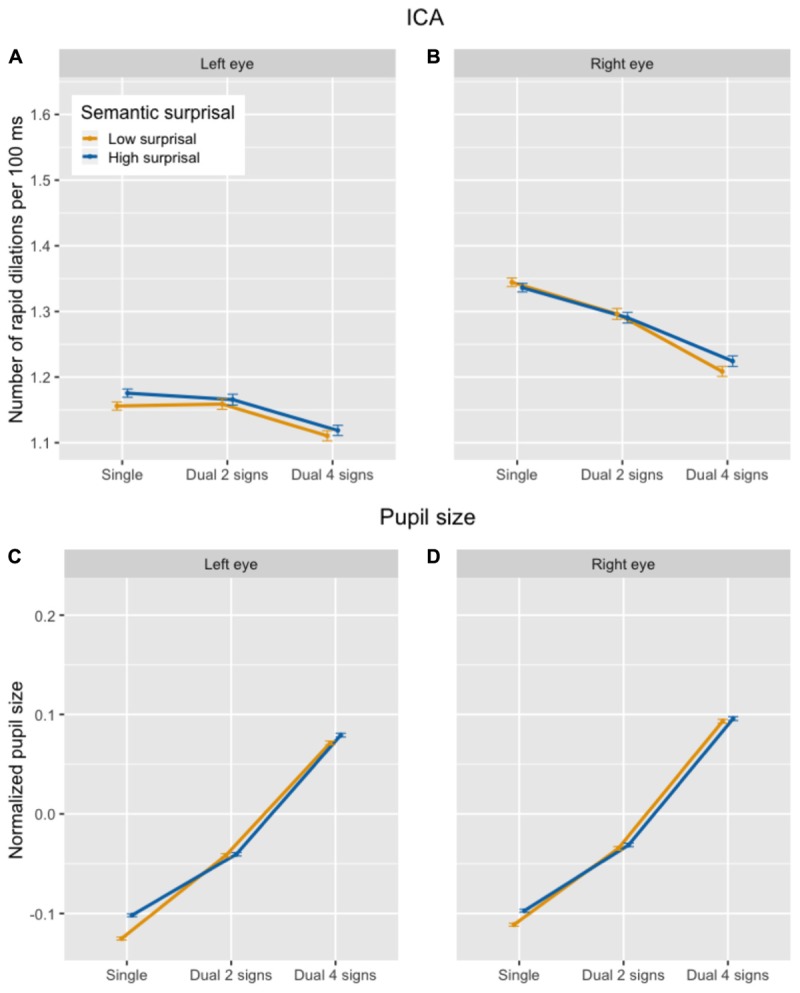
Number of ICA events per 100 ms **(A,B)** and overall pupil size normalized across participants **(C,D)** in the listening task, by secondary task difficulty and semantic surprisal condition.

There was no statistically significant difference in the ICA between the single and the dual task blocks. However, within the dual task we found a significantly *lower* frequency of rapid pupil dilations in the difficult (4 signs) than in the easy (2 signs) memory task (see top half of Figure 2). The main effect of the control factor semantic surprisal was not statistically significant over the listening blocks as a whole. As we will see in section “Stimulus-Evoked Load Analysis,” however, semantic surprisal is a significant predictor in our analysis of the ICA in response to our specific stimuli. Including the interaction between semantic surprisal and secondary task difficulty did not significantly contribute to model fit [χ^2^(2) = 1.20; *p* = 0.55], so it was taken out of the model. Table 1A presents the output of the final mixed-effects model.

**Table 1:** Poisson mixed model output for the ICA **(A)** and linear mixed model output for overall pupil size **(B)** in the listening task blocks.

	Coef.	*SE*	*z*-value	*p*-value	95% CI
**A**					
Intercept	0.0043	0.0872	0.0489	0.9610	[-0.167, 0.175]
Semantic surprisal	0.0126	0.0178	0.7063	0.4800	[-0.022, 0.047]
Single vs. dual task	0.0113	0.0358	0.3152	0.7526	[-0.059, 0.081]
Easy vs. difficult memory task	-0.0615	0.0237	-2.5950	0.0095^∗∗^	[-0.108, -0.015]
*Model specification: ICA ∼ surp + taskdif + (1 + surp ^∗^ sing/dual + surp ^∗^ eas/dif || item) + (1 + surp + sing/dual + surp ^∗^ eas/dif ||participant) + (1|participant:eye)*					

**B**					
Intercept	-0.0200	0.0146	-1.3745	0.1755	[-0.049, 0.009]
Semantic surprisal	0.0087	0.0141	0.6153	0.5406	[-0.019, 0.036]
Single vs. dual task	0.1261	0.0367	3.4377	0.0015^∗∗^	[0.054, 0.198]
Easy vs. difficult memory task	0.1249	0.0245	5.1094	<0.001^∗∗∗^	[0.077, 0.173]
*Model specification: pupilsize ∼ surp + taskdif + (1 + surp ^∗^ sing/dual + surp ^∗^ eas/dif || item) + (1 + surp + sing/dual + surp ^∗^ eas/dif||participant) + (1|participant:eye)*					


*^∗∗^*p* < 0.01; ^∗∗∗^*p* < 0.001.*

##### Pupil size

For overall pupil size, the effect of secondary task difficulty was statistically significant, with larger pupil sizes in the dual task than in the single task. In addition, the difficult memory task (4 signs) condition showed a significantly *larger* pupil size than the easy memory task (2 signs) condition, in contrast with the ICA (see bottom half of Figure 2). Again, there was no statistically significant difference between the low- and high-surprisal conditions measured over the listening blocks as a whole. Including the interaction between semantic surprisal and secondary task difficulty did not significantly contribute to model fit [χ^2^(2) = 0.43; *p* = 0.81], so it was taken out of the model. The output in Table 1B shows the final mixed-effects model.

##### Task performance

Accuracy on the comprehension questions was generally high (92.9% correct on average). None of the main effects were statistically significant. However, there was a statistically significant interaction between semantic surprisal and single vs. dual task, suggesting that accuracy was lower in high- as compared to low-surprisal sentences, but only in the single task. Table 2A shows the output of the first converging mixed-effects model.

**Table 2:** Logit mixed model output for comprehension question accuracy **(A)** and logit mixed model output for sign recall accuracy **(B)** in the listening task blocks.

	Coef.	*SE*	*z*-value	*p*-value	95% CI
**A**					
Intercept	3.3134	0.3856	8.5927	<0.001^∗∗∗^	[2.558, 4.069]
Semantic surprisal	-0.0816	0.3930	-0.2076	0.8355	[-0.852, 0.689]
Single vs. dual task	-0.7862	0.6160	-1.2764	0.2018	[-1.994, 0.421]
Easy vs. difficult memory task	-0.0509	0.5265	-0.0968	0.9229	[-1.083, 0.981]
Semantic surprisal : single vs. dual task	1.5827	0.7939	1.9936	0.0462^∗^	[0.027, 3.139]
Semantic surprisal : easy vs. difficult memory task	-1.4194	1.0608	-1.3380	0.1809	[-3.499, 0.660]
*Model specification: Qacc ∼ surp ^∗^ taskdif + (1|participant) + (1 + sing/dual ||item)*					

**B**					
Intercept	1.8790	0.1441	13.0378	<0.001^∗∗∗^	[1.597, 2.161]
Semantic surprisal	-0.2369	0.2297	-1.0314	0.3024	[-0.687, 0.213]
Easy vs. difficult memory task	-0.0356	0.2290	-0.1552	0.8766	[-0.484, 0.413]
*Model specification: Signacc ∼ surp + eas/dif + (1 + eas/dif ||participant) + (1|item)*					


*^∗^*p* < 0.05; ^∗∗∗^*p* < 0.001.*

The proportion of correctly recalled series of speed limit signs was also high (86.0% correct on average). Note that we used quite a strict measure for recall accuracy here, as all signs in a series had to be recalled correctly in the correct order. Recalling all signs but one correctly or swapping around two signs was thus counted as incorrect. Neither semantic surprisal nor secondary task difficulty had a statistically significant effect on recall accuracy. The interaction term did not significantly improve model fit [χ^2^(1) = 0.16; *p* = 0.69], so it was removed from the model. Table 2B shows the output of the first converging mixed-effects model.

#### Driving Task

##### Index of cognitive activity (ICA)

**FIGURE 3:**
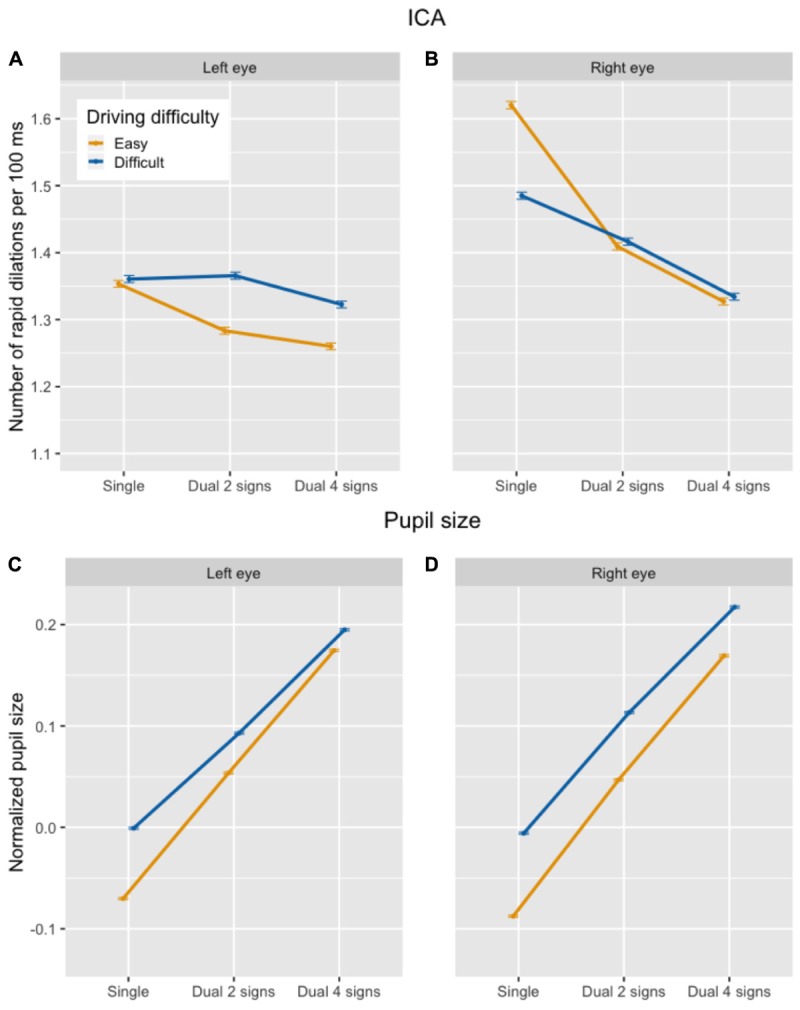
Number of ICA events per 100 ms **(A,B)** and overall pupil size normalized across participants **(C,D)** in the driving task, by secondary task difficulty and driving difficulty condition.

In the driving task, there was a statistically significant effect of secondary task difficulty on the ICA, with the frequency of rapid pupil dilations being *lower* in the dual task as compared to the single task, and in the difficult (4 signs) as compared to the easy (2 signs) memory task (see top half of Figure 3), contrary to what would be predicted if the ICA increased with increased cognitive load. In line with earlier studies using the same driving paradigm, there was a main effect of driving difficulty, with more rapid dilations in the difficult than in the easy driving task. However, there was also a statistically significant interaction between driving difficulty and single vs. dual task. Paired comparisons showed that the decrease in ICA between the easy (2 signs) and the difficult (4 signs) secondary task was statistically significant in both the easy and the difficult driving task, but there was only a significant decrease between the single and the dual task in the easy driving task, and not in the difficult driving task. Table 3A shows the output of the final mixed-effects model.^7^

**Table 3:** Poisson mixed model output for the ICA **(A)** and linear mixed model output for overall pupil size **(B)** in the driving task blocks.

	Coef.	*SE*	*z*-value	*p*-value	95% CI
**A**					
Intercept	0.1198	0.0400	2.9962	0.0027^∗∗^	[0.041, 0.198]
Driving difficulty	0.0065	0.0017	3.7739	<0.001^∗∗∗^	[0.003, 0.010]
Single vs. dual task	-0.0649	0.0252	-2.5685	0.0102^∗^	[-0.114, -0.015]
Easy vs. difficult memory task	-0.0359	0.0101	-3.5742	<0.001^∗∗∗^	[-0.056, -0.016]
Driving difficulty : single vs. dual task	0.0805	0.0036	22.4917	<0.001^∗∗∗^	[0.074, 0.088]
Driving difficulty : easy vs. difficult memory task	-0.0033	0.0043	-0.7725	0.4398	[-0.012, 0.005]
*Model specification: ICA ∼ drivdif ^∗^ taskdif + (1 + sing/dual + eas/dif*participant) + (1|participant:eye)**					

**B**					
Intercept	0.0748	0.0158	4.7424	<0.001^∗∗∗^	[0.044, 0.106]
Driving difficulty	0.0513	0.0346	1.4814	0.1506	[-0.017, 0.119]
Single vs. dual task	0.1747	0.0234	7.4739	<0.001^∗∗∗^	[0.129, 0.220]
Easy vs. difficult memory task	0.1135	0.0154	7.3618	<0.001^∗∗∗^	[0.083, 0.144]
*Model specification: pupilsize ∼ drivdif + taskdif + (1 + drivdif ^∗^ sing/dual + drivdif ^∗^ eas/dif*participant) + (1|participant:eye)**					


*^∗^*p* < 0.05; ^∗∗^*p* < 0.01; ^∗∗∗^*p* < 0.001.*

##### Pupil size

In contrast to the ICA, there was a statistically significant *increase* in overall pupil size between the single and dual task and between the easy (2 signs) and difficult (4 signs) memory task (see bottom half of Figure 3), as predicted. The main effect of driving difficulty was not statistically significant. The interaction between driving difficulty and secondary task difficulty did not significantly improve model fit [χ^2^(2) = 4.52; *p* = 0.10] and was taken out of the model. The output of the final model is presented in Table 3B.

##### Task performance

There were statistically significant main effects of both secondary task difficulty and driving difficulty on driving performance: steering deviations were larger (worse performance) in difficult as compared to easy driving, and also increased with increased memory task difficulty. Including the interaction between driving difficulty and secondary task difficulty did not significantly improve model fit [χ^2^(2) = 3.71; *p* = 0.16], so it was dropped from the model. The output of the maximal model is presented in Table 4A.

**Table 4:** Linear mixed model output for steering performance **(A)** and logit mixed model output for sign recall accuracy **(B)** in the driving task.

	Coef.	*SE*	*z*-value	*p*-value	95% CI
**A**					
Intercept	0.4911	0.0182	27.0546	<0.001^∗∗∗^	[0.456, 0.527]
Driving difficulty	0.3316	0.0154	21.5055	<0.001^∗∗∗^	[0.301, 0.362]
Single vs. dual task	0.0298	0.0047	6.3863	<0.001^∗∗∗^	[0.021, 0.039]
Easy vs. difficult memory task	0.0252	0.0041	6.1844	<0.001^∗∗∗^	[0.017, 0.033]
*Model specification: steerperf ∼ drivdif + taskdif + (1 + drivdif ^∗^ eas/dif|participant)*					

**B**					
Intercept	2.8751	0.1467	19.5931	<0.001^∗∗∗^	[2.588, 3.163]
Driving difficulty	-0.2353	0.2074	-1.1346	0.2565	[-0.642, 0.171]
Easy vs. difficult memory task	-1.1232	0.2222	-5.0558	<0.001^∗∗∗^	[-1.559, -0.688]
*Model specification: signacc ∼ drivdif + eas/dif + (1 + drivdif ^∗^ eas/dif ||participant)*					


*^∗∗∗^*p* < 0.001.*

Recall accuracy for the speed limit signs was high (93.3% correct on average). There was a significant effect of secondary task difficulty on recall accuracy: the proportion of correctly recalled signs was lower in the difficult (4 signs) than in the easy (2 signs) memory task. The main effect of driving difficulty was not statistically significant. Including the interaction term in the model did not improve model fit [χ^2^(1) = 1.22; *p* = 0.27], and it was taken out of the model. The output of the first converging model is shown in Table 4B. Note that memory task performance was much higher in the dual task setting with the driving task compared to the dual task setting where language comprehension was used as a primary task. This indicates that participants allocated more cognitive resources to the language comprehension task than to the driving task.

##### Summary single vs. dual task analysis

In the listening task, accuracy on the comprehension questions was high, suggesting that participants were paying attention to the linguistic stimuli. The recall of the speed limit signs was also good, although much lower than in the dual task with driving, suggesting that the listening task interfered more with the secondary memory task. In the single task, comprehension question accuracy was lower on the high- than on the low-surprisal sentences, consistent with the assumption that the high-surprisal items were more difficult. The fact that in the dual task neither semantic surprisal nor secondary task difficulty affected task performance might imply that, under the pressure of the dual task, participants had recruited additional cognitive resources to retain a similar performance level. Consistent with this interpretation, overall pupil size was greater in the dual task as compared to the single task, as well as in the difficult as compared to the easy memory task condition. Participants thus seem to have maintained their task effectiveness at the cost of less efficient use of cognitive resources. The ICA, on the other hand, showed no increase in the frequency of rapid dilations in the dual task as compared to the single task, and even a decrease in the difficult memory task as compared to the easy memory task, suggesting that the ICA is not sensitive to an increase in cognitive load induced by performing multiple tasks at the same time.

In the driving task, steering performance decreased in the difficult as compared to the easy driving task, and was also lower in the dual task than in the single task, with further deteriorated performance in the more difficult memory task (recalling 4 speed limit signs rather than 2), as expected. The recall accuracy of the speed limit signs was also lower in the more difficult memory task condition. This confirms that the conditions that were designed to be difficult were indeed more difficult. The dual task showed larger overall pupil sizes compared to single task driving, and more difficult dual tasking requiring the recall of 4 speed limit signs also showed an increase in pupil size as compared to recalling 2 speed limit signs. However, the ICA significantly *decreased* in the 4 speed limit signs condition as compared to the 2 signs condition. Similarly, the ICA was lower in the dual than in the single task, although this effect was modulated by an interaction with driving difficulty, with the difference only being statistically significant in the easy driving condition. These findings further support the differential sensitivity of the two pupillometric measures to single vs. dual task difficulty manipulations, and show that this is not restricted to specific combinations of task domains.

### Stimulus-Evoked Load Analysis

#### Listening Task

##### Index of cognitive activity (ICA)

**FIGURE 4:**
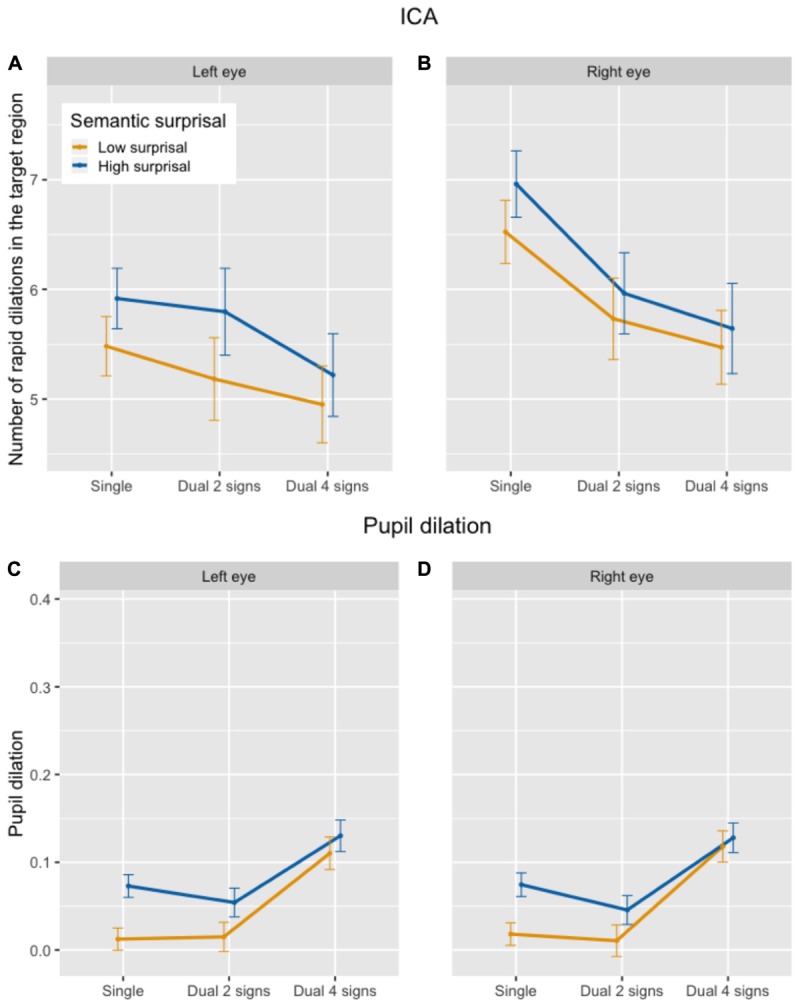
Number of ICA events in the target region **(A,B)** and average pupil dilation in the target region **(C,D)** in the listening task, by semantic surprisal and secondary task difficulty.

There was a statistically significant effect of semantic surprisal on the ICA in the listening task, with a higher frequency of rapid pupil dilations following the presentation of a high-surprisal word as compared to a low-surprisal word, as predicted (see top half of Figure 4). The main effects of the factors for secondary task difficulty, which acted as control factors for this analysis, were not significant. Including the interaction term did not significantly contribute to model fit [χ^2^(2) = 2.50; *p* = 0.29], and was therefore taken out of the model. Table 5A presents the output of the final mixed-effects model.

**Table 5:** Poisson mixed model output for the ICA **(A)** and linear mixed model output for pupil dilation **(B)** in the target region in the listening task.

	Coef.	*SE*	*z*-value	*p*-value	95% CI
**A**					
Intercept	1.5344	0.1129	13.5947	<0.001^∗∗∗^	[1.313, 1.756]
Semantic surprisal	0.0656	0.0325	2.0208	0.0433^∗^	[0.002, 0.129]
Single vs. dual task	-0.0864	0.0561	-1.5409	0.1233	[-0.196, 0.024]
Easy vs. difficult memory task	-0.0712	0.0550	-1.2948	0.1954	[-0.179, 0.037]
*Model specification: ICA ∼ surp + taskdif + (1 + surp + sing/dual + surp ^∗^ eas/dif ||item) + (1 + surp ^∗^ sing/dual + surp ^∗^ eas/dif ||participant) + (1|participant:eye)*					

**B**					
Intercept	0.0693	0.0087	7.9533	<0.001^∗∗∗^	[0.052, 0.086]
Semantic surprisal	0.0408	0.0162	2.5172	0.0161^∗^	[0.009, 0.073]
Single vs. dual task	0.0345	0.0179	1.9327	0.0601	[0.000, 0.070]
Easy vs. difficult memory task	0.0912	0.0242	3.7731	<0.001^∗∗∗^	[0.044, 0.139]
*Model specification: pupildilation ∼ surp + taskdif + (1 + surp ^∗^ sing/dual + surp ^∗^ eas/dif || item) + (1 + surp ^∗^ sing/dual + surp ^∗^ eas/dif ||participant) + (1|*					
*participant: eye)*					


*^∗^*p* < 0.05; ^∗∗∗^*p* < 0.001.*

##### Pupil dilation

The effect of semantic surprisal on pupil dilation was statistically significant, with larger pupil dilations following the presentation of a high-surprisal word as compared to a low-surprisal word, in line with the ICA (see bottom half of Figure 4). In contrast with the ICA, however, within the dual task the difficult memory task (4 signs) condition showed a significantly larger pupil dilation in the target region than the easy memory task (2 signs) condition. Including the interaction term did not significantly improve model fit [χ^2^(2) = 2.01; *p* = 0.37] and it was dropped from the model. The output in Table 5B shows the final mixed-effects model.

#### Memory Task

##### Index of cognitive activity (ICA)

**FIGURE 5:**
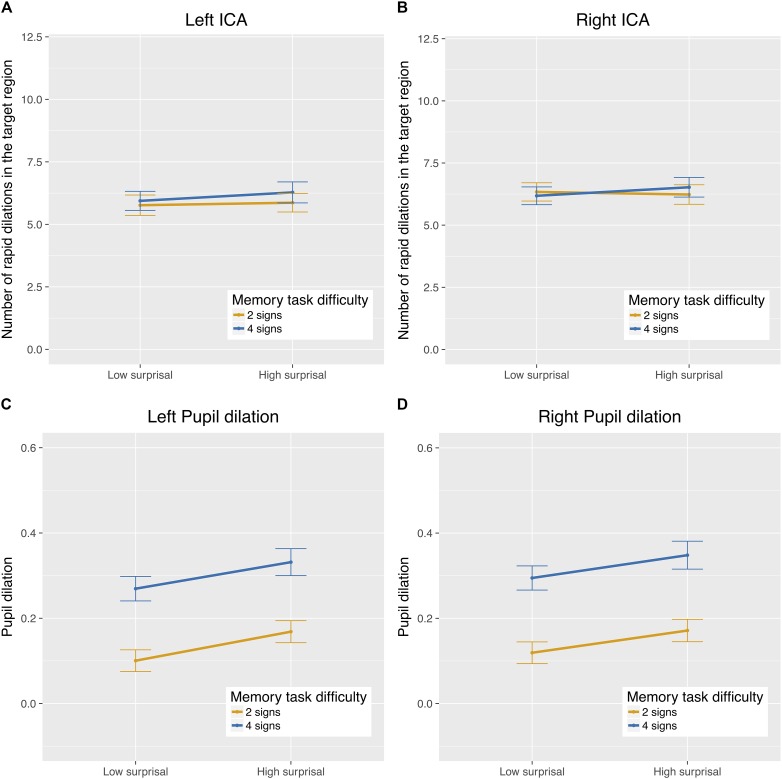
Number of ICA events in the target region **(A,B)** and average pupil dilation in the target region **(C,D)** in the memory + listening task, by memory task difficulty and semantic surprisal.

In the listening task, there was no statistically significant effect of memory task difficulty on the ICA in the region following the presentation of the final speed limit sign (see top half of Figure 5). In addition, there was no main effect of semantic surprisal. The interaction did not significantly improve model fit [χ^2^(1) = 0.66; *p* = 0.42] and was taken out of the model. The output of the final mixed-effects model is presented in Table 6A.

**Table 6:** Poisson mixed model output for the ICA **(A)** and linear mixed model output for pupil dilation **(B)** in the target region in the memory+listening task.

	Coef.	*SE*	*z*-value	*p*-value	95% CI
**A**					
Intercept	1.6197	0.1106	14.6393	<0.001^∗∗∗^	[1.403, 1.837]
Semantic surprisal	0.0133	0.0410	0.3252	0.7450	[-0.067, 0.094]
Easy vs. difficult memory task	0.0196	0.0489	0.4005	0.6888	[-0.076, 0.115]
*Model specification: ICA ∼ surp + eas/dif + (1 + surp ^∗^ eas/dif ||item) + (1 + surp ^∗^ eas/dif || participant) + (1|participant:eye)*					

**B**					
Intercept	0.2369	0.0372	6.3601	<0.001^∗∗∗^	[0.164, 0.310]
Semantic surprisal	0.0609	0.0259	2.3555	0.0233^∗^	[0.010, 0.112]
Easy vs. difficult memory task	0.1696	0.0365	4.6421	<0.001^∗∗∗^	[0.098, 0.241]
*Model specification: pupildilation ∼ surp + eas/dif + (1 + surp ^∗^ eas/dif|item) + (1 + surp ^∗^ eas/dif| participant) + (1|participant:eye)*					


*^∗^*p* < 0.05; ^∗∗∗^*p* < 0.001.*

**FIGURE 6:**
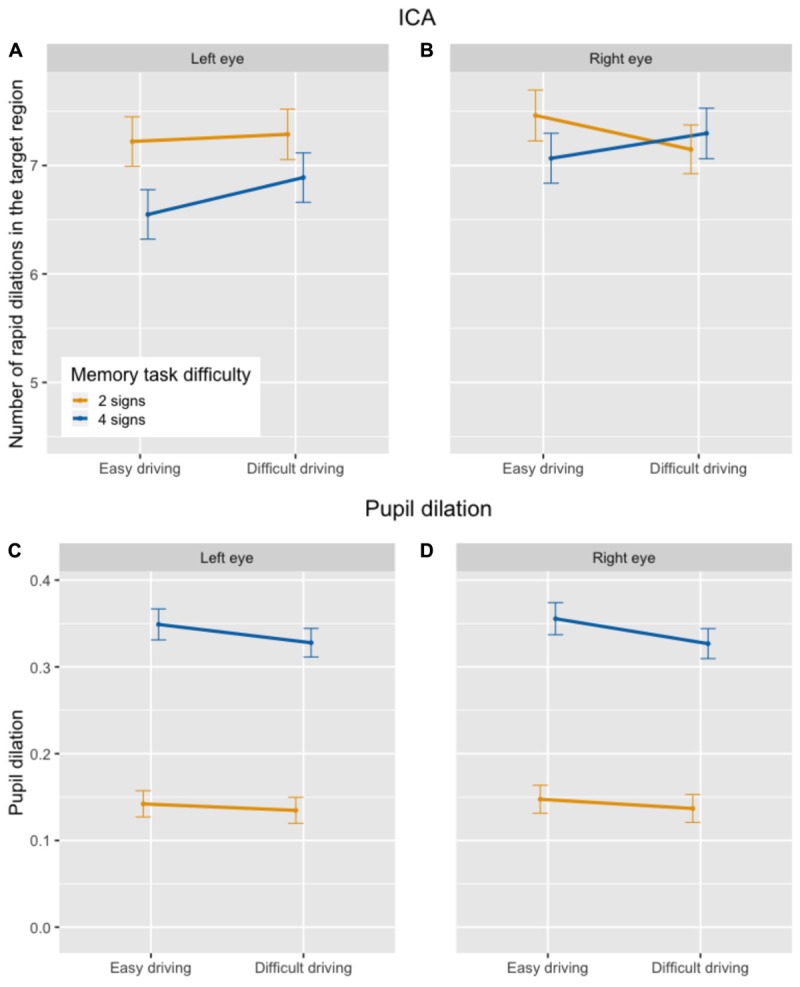
Number of ICA events in the target region **(A,B)** and average pupil dilation in the target region **(C,D)** in the memory + driving task, by memory task difficulty and driving difficulty.

In the driving task, although the frequency of rapid pupil dilations seemed somewhat *lower* in the difficult (4 signs) than in the easy (2 signs) memory task (see top half of Figure 6), there was no statistically significant effect of memory task difficulty on the ICA in the region following the presentation of the final speed limit sign. In addition, there was no main effect of driving difficulty, and no interaction. The output of the maximal mixed-effects model is presented in Table 7A.

**Table 7:** Poisson mixed model output for the ICA **(A)** and linear mixed model output for pupil dilation **(B)** in the target region in the memory+driving task.

	Coef.	*SE*	*z*-value	*p*-value	95% CI
**A**					
Intercept	1.8129	0.1010	17.9564	<0.001^∗∗∗^	[1.615, 2.011]
Driving difficulty	0.0138	0.0355	0.3881	0.6979	[-0.056, 0.083]
Easy vs. difficult memory task	-0.0396	0.0262	-1.5095	0.1312	[-0.091, 0.012]
Driving difficulty : easy vs. difficult memory task	0.0586	0.0338	1.7360	0.0826	[-0.008, 0.125]
*Model specification: ICA ∼ drivdif ^∗^ eas/dif + (1 + drivdif ^∗^ eas/dif|participant) + (1| participant:eye)*					

**B**					
Intercept	0.2407	0.0337	7.1448	<0.001^∗∗∗^	[0.175, 0.307]
Driving difficulty	-0.0129	0.0246	-0.5254	0.6039	[-0.061, 0.035]
Easy vs. difficult memory task	0.2057	0.0277	7.4388	<0.001^∗∗∗^	[0.152, 0.260]
*Model specification: pupilsize ∼ drivdif + eas/dif + (1 + drivdif ^∗^ eas/dif|participant) + (1 | participant:eye)*					


*^∗∗∗^*p* < 0.001.*

##### Pupil size

In the listening task, there was a statistically significant increase in pupil dilation between easy (2 signs) and difficult (4 signs) dual tasking in the region following the presentation of the last speed limit sign (see bottom half of Figure 5), in contrast to the ICA. In addition, there was a statistically significant main effect of semantic surprisal, with larger pupil dilations in trials containing a surprising word. The interaction between semantic surprisal and easy vs. difficult memory task did not significantly improve model fit [χ^2^(1) = 0.64; *p* = 0.42] and was taken out of the model. The output of the maximal model is presented in Table 6B.

In the driving task, there was also a significant increase in pupil dilation between easy (2 signs) and difficult (4 signs) dual tasking in the region following the presentation of the last speed limit sign (see bottom half of Figure 6). The main effect of driving difficulty was not statistically significant. The interaction between driving difficulty and easy vs. difficult memory task did not significantly improve model fit [χ^2^(1) = 0.13; *p* = 0.71] and was taken out of the model. The output of the maximal model is presented in Table 7B.

##### Summary stimulus-evoked load analysis

In our stimulus-evoked load analysis, we found that ICA events were more frequent after highly surprising words than after predictable words, in line with the increase in pupil dilations found for high-surprisal words, and hence supporting the ICA as a measure of increased stimulus-evoked cognitive load. Although the surprisal effect seemed to become smaller in more difficult dual tasking, the interaction between semantic surprisal and memory task difficulty was not statistically significant, suggesting that the surprisal effect can still be detected in difficult dual tasks. The fact that the surprisal effect on pupil dilations, but not on the ICA, remained present after the presentation of the final speed limit sign is an indication of the more dynamic nature of the ICA. Pupil dilations were also larger in the difficult memory task (4 signs) as compared to the easy memory task (2 signs), both in the target word region and in the final sign presentation region. The ICA, on the other hand, showed no reliable increase in the frequency of rapid dilations with a more difficult memory task, neither in the target word region, nor in the final sign presentation region. This suggests that the ICA is not sensitive to an increase in cognitive load induced by a secondary WM task.

## Discussion

In this study, we systematically investigated the Index of Cognitive Activity as a measure of processing load in two previously untested dual-task settings: language comprehension combined with a memory task, and simulated driving combined with the same memory task. Based on previous research, we predicted that the frequency of ICA events (rapid pupil dilations) would increase with surprising as compared to predictable words, provided that they are sufficiently attended. We also predicted that the ICA would increase in a difficult compared to an easy driving task. More importantly, if the ICA is, as claimed, an indicator of cognitive load, similar to, but more dynamic than pupil size, it should also show an increase when participants need to perform two tasks at the same time, as compared to performing a single task. On the other hand, if the ICA and pupil size are measures that reflect different cognitive processes with different neuronal underpinnings, they need not respond similarly to increases in cognitive load. Thus, we compared the ICA response in the dual-task settings to overall pupil size and stimulus-evoked pupil dilation, as well as to task performance. We expected larger pupil dilations in response to the more difficult listening, driving, and memory task conditions as compared to the easy conditions, as well as larger overall pupil size and lower performance in dual- as compared to single-task settings.

Our results support the idea that the ICA and the size of the pupil measure different underlying cognitive processes. In the listening task, even though we found that overall pupil size was larger in the dual than in the single task, as well as in the more difficult memory task (recalling 4 speed limit signs) as compared to the easier memory task (recalling 2 signs), the ICA did not show an increase with an increased situational demand due to dual tasking. In fact, within the dual task the frequency of rapid dilations was significantly *lower* in the more difficult memory task as compared to the easier memory task. Similarly, in the simulated driving task overall pupil size was greater in dual-task than single-task driving, and also increased with higher memory load (4 vs. 2 signs). The ICA, by contrast, showed a *decrease* in dual-task as compared to single-task driving, at least in the easy driving task, as well as a decrease with higher memory load. Our two dual task combinations thus show consistent results: increases in pupil size but no increase in the ICA in a (difficult) dual task as compared to a single task. These findings are in line with Demberg et al. (2013b), who also found a (significant) decrease instead of an increase in the frequency of rapid pupil dilations in a driving and listening dual task as compared to driving only.

At the same time, words that were unexpected (and hence difficult to process) showed more frequent rapid pupil dilations than predictable words in the listening task, supporting the ICA as a measure of increased cognitive load in language processing (cf. Demberg and Sayeed, 2016). In fact, the semantic surprisal effect was similar to the effect of semantic violations found previously (Demberg et al., 2013a; Demberg and Sayeed, 2016). The pupil dilation measure showed a similar effect of semantic surprisal, suggesting that the two pupillometry measures are both sensitive to increased cognitive load evoked by an unexpected stimulus. By contrast, only pupil dilation showed an effect of the speed limit sign manipulation (2 vs. 4 signs) in the dual tasks in the target region following the presentation of the last sign. The ICA did not show a difference between the 2- and 4-sign conditions in the same region, suggesting that the ICA is not sensitive to increases in WM load when attention is already spread over two tasks in the dual task setting.

In the driving task, driving difficulty influenced the ICA, but not consistently across the single and the dual task. Overall pupil size did not show a statistically significant effect of driving difficulty. The fact that we did not replicate the previously found pupillometric effect of the driving difficulty manipulation (cf. Demberg, 2013; Demberg et al., 2013b; Engonopoulos et al., 2013; Demberg and Sayeed, 2016) might be due to differences in the driving task settings (driving speed, lateral speed of the reference bar and maximum speed of the steering bar). Our settings differed slightly from those in the driving studies cited above, to accommodate the combination with the secondary task. It might be the case that this caused the easy and difficult driving conditions to be too similar to induce a difference in mental workload.

As for task performance, accuracy on the comprehension questions and on the speed limit sign recall in the listening task was generally high, indicating that participants were paying attention to the linguistic materials as well as to the memory task. Accuracy on the comprehension questions decreased with higher surprisal in the single task, but was not affected by the addition of the secondary task. In addition, there were no effects of either surprisal or memory task difficulty on performance in the dual task. These findings suggest that participants were able to maintain good performance on both tasks in the dual task, but at the cost of increased arousal, as evidenced by the greater overall pupil size in the dual task. Thus, there seems to be a trade-off between the effectiveness (the quality of performance) and the efficiency (performance in relation to the effort put into it) of participants’ task performance (Kahneman, 1973; Karatekin et al., 2004). In the simulated driving task, by contrast, steering performance declined both with a higher driving difficulty and with the addition of the secondary memory task, as well as with an increase in memory load. Sign recall accuracy was also affected by the memory task difficulty. This confirms that the driving and memory dual task was more difficult than single driving, and suggests that the task difficulty could not be fully compensated by allocating additional cognitive resources to the tasks in this case.

In sum, the results of the present study have shown that increasing cognitive load by the addition of a secondary task, although leading to an increase in pupil size, need not increase, and may even decrease, the frequency of rapid pupil dilations. We found evidence for this in both a listening and memory dual task and a driving and memory dual task, suggesting that there is a general dissociation between pupil size and the ICA, which is apparent across verbal and non-verbal tasks and is not restricted to a particular type of task combination.

Given these findings, and given that pupil size and the ICA are both considered measures of cognitive load, the question arises how the dissociation between the two measures can be explained within a single definition of cognitive load. One potential answer may lie in the two modes of brain activity in the LC area. As we have seen, rapid increases in pupil size are considered to be closely related to high phasic activity in the LC, and hence can be associated with task engagement and the recruitment of additional attention resources in response to salient stimuli relevant to that task (cf. Gilzenrat et al., 2010; Murphy et al., 2011; Laeng et al., 2012; Alnæs et al., 2014). Since the ICA is based on the frequency of these rapid pupil dilations, it should especially correspond to phasic LC activity. Because the ICA has a low auto-correlation, it is a highly dynamic measure that is closely aligned with a person’s response to a stimulus (Demberg and Sayeed, 2016). As soon as the stimulus has been responded to, the ICA will decrease again. Hence, the ICA should be especially sensitive to the momentary increases in attention allocation in response to salient and task-relevant stimuli. In line with the Adaptive Gain Theory, the frequency of ICA events should then be high when task engagement is high, and lower in cases of divided attentional focus. One situation that is likely to cause a spread of focus is the addition of a (difficult) secondary task, requiring attention to be distributed over multiple tasks. Thus, this argument might explain why the ICA did not increase in our dual tasks as compared to the single tasks, and even decreased in some cases. In this view, a decrease in the frequency of ICA events in dual tasking signifies the cognitive adaptation to a new task demand where attentional focus needs to be distributed over more than one task.

The conventional pupil size measure, by contrast, is highly correlated with itself on consecutive time points (cf. Demberg et al., 2013b) due to physical restrictions on how rapidly the overall pupil area can increase (dilation) and decrease (constriction). Therefore, it is a less dynamic measure than the ICA. An increased pupil size will be sustained after attention to the stimulus has faded or the task has been solved. In contrast to the ICA, then, pupil size might also partly relate to the degree of tonic LC activity, and reflect the cumulated arousal associated with a new, more demanding task environment. This would be in line with the finding in our experiment that both overall pupil size and stimulus-evoked pupil dilations became larger with more difficult dual tasking, and also conforms to earlier findings of larger pupil size increases in more difficult dual tasking (e.g., Hyönä et al., 1995; Karatekin et al., 2004; Koelewijn et al., 2014).

Based on these results, we propose that the ICA measures a different underlying cognitive process than pupil size, and we conjecture that this difference may relate to the phasic and tonic modes of neuronal activity in the LC. Future research should explicitly test this hypothesis. One way to monitor the ICA in relation to changing task demands is to investigate individual differences in task performance and cognitive load, which could potentially reveal a more complex interaction between task difficulty and individual task strategies or cognitive capacities in the ICA effects. One prediction of the Adaptive Gain Theory is that the use of additional resources as indexed by rapid pupil dilations will lead to better task performance. Participants with high performance would thus be likely to have generally higher ICA values than low-performing participants. A *post hoc* analysis grouping the participants in our data by task performance using a median split seems to support this prediction for single task driving (but not for single task listening): relatively good drivers had an average of 1.409 (95% CI: 1.405–1.414) rapid dilations per 100 ms, whereas relatively poor drivers had an average of 1.347 (95% CI: 1.343–1.352) rapid dilations per 100 ms. In addition, in cases of dual tasking in which focus has to continuously switch between different tasks, a tonic mode might actually lead to higher performance than a phasic mode (cf. Koelewijn et al., 2014). In dual-task conditions, a lower ICA should then correspond to a better task-switching ability, and hence to reasonable performance on both tasks (as opposed to good performance on one task but not on the other, or poor performance overall). This prediction is borne out in the *post hoc* analysis: In dual task driving, participants with relatively good performance on both tasks had a substantially lower average ICA (1.037; 95% CI: 1.033–1.042) than participants prioritizing one task (1.591; 95% CI: 1.587–1.596) or participants with relatively poor performance on both tasks (1.364; 95% CI: 1.359–1.370). The same was true for dual task listening: Participants with good performance on both tasks had a lower ICA (0.931; 95% CI: 0.925–0.937) than participants prioritizing one task (1.333; 95% CI: 1.326–1.341) or participants with relatively poor performance on both tasks (1.360; 95% CI: 1.352–1.368). Still, individual differences in the frequency of ICA events and their interaction with task difficulty manipulations need to be investigated more systematically, preferably using participant groups that are expected to show large differences in task performance and cognitive capacity. We are currently extending our research to elderly adults (see Häuser et al., 2017), which allows us to investigate a broader range of cognitive capacities, and hence to get a better picture of the effect of cognitive capacity on processing difficulty.

## Conclusion

This study supports the ICA as a measure of cognitive load invoked by linguistic stimuli. More importantly, however, we have shown across different dual-task situations that the ICA differs from the conventional pupil size measure in cases of increased situational demand: whereas larger pupil sizes and larger pupil dilations were found with more difficult dual tasking, the frequency of ICA events did not increase or even decreased in the same conditions. We contribute this difference to the dynamic nature of the ICA, which makes it sensitive to cognitive adaptations to new task demands: when attentional resources need to be distributed over multiple tasks, this leads to a decrease in the frequency of rapid pupil dilations. Our findings thus suggest that the ICA is a sensitive measure of the degree of stimulus-evoked cognitive load, and could therefore complement overall pupil dilation as a measure of cognitive load in tasks involving rapid succession of events and/or overlapping stimuli.

## Data Availability

The datasets generated for this study can be found in the Open Science Framework (OSF) repository for this paper: https://osf.io/mjpqn/?view_only=c26f1202f2934cebb340f3d4396c791f.

## Author Contributions

JV, VD, and JK conceived of this study. JV conducted the research and wrote the article. VD and JK provided feedback on the research process and on an earlier version of this article.

## Conflict of Interest Statement

The authors declare that the research was conducted in the absence of any commercial or financial relationships that could be construed as a potential conflict of interest.

## Footnotes

1 Also note that even in constant lighting conditions, the pupil oscillates randomly, causing a potential confound for pupillometric measures of cognitive load.

2 opends.de

3 sr-research.com

4 mary.dfki.de

5 During the experiment, the relative clauses turned out to be too hard to process in this particular task setting. Therefore, we only report results on our semantic manipulation here.

6 Due to updates to the Cognitive Workload Module software, there are differences between the frequency and latency of ICA events reported here and those reported in earlier studies. A reanalysis of the data from Demberg and Sayeed (2016) using the new version of the software yielded virtually the same effects.

7 This model still gave a non-convergence warning in R, but running the same model with different optimizers showed stable estimates, so we considered the warning a false positive.

## APPENDIX

**Table A1:** Psychometric tests conducted after the main experiment.

Test	Measure of	Mean scoreand range
Counting Span test (Engle et al., 1999)	WM capacity	27.9 (15–51); max: 54
Digit-Symbol test (Wechsler, 1981)	Perceptual speed of processing	66.2 (42–89); max: 90
